# Off-Label Pediatric Medication Prescribing and Dispensing: Awareness and Attitudes among Community Pharmacists: A Questionnaire-Based Study

**DOI:** 10.3390/pharmacy12050149

**Published:** 2024-09-27

**Authors:** Carmen-Maria Jîtcă, George Jîtcă, Imre Silvia

**Affiliations:** 1 Doctoral School of Medicine and Pharmacy, Institution Organizing Doctor’s Degree University Studies, George Emil Palade University of Medicine, Pharmacy, Science and Technology of Târgu Mureș, 540139 Targu Mures, Romania; carmenrusz20@gmail.com; 2 Department of Pharmacology and Clinical Pharmacy, Faculty of Pharmacy, George Emil Palade University of Medicine, Pharmacy, Science and Technology of Târgu Mureș, 540139 Targu Mures, Romania; 3 Department of Analytical Chemistry and Drug Analysis, Faculty of Pharmacy, ‘George Emil Palade’ University of Medicine, Pharmacy, Science, and Technology of Targu Mures, 540142 Targu Mures, Romania; silvia.imre@umfst.ro

**Keywords:** off label, community pharmacy, survey, pediatrics, pharmacy

## Abstract

Off-label practice in pediatrics requires relentless engagement from all the health professionals involved. Community pharmacists are the last ones in the prescribing–dispensing chain; therefore, they have the key responsibility of verifying the correctness of a treatment. A cross-sectional study was conducted for assessing the awareness and views of Romanian community pharmacists, regarding off-label drugs in the pediatric population, through a 28-item questionnaire comprising five sections of different topics (general knowledge, frequency of prescribing and dispensing off-label medication, views, and attitudes). The sample size was 236 questionnaires with a response rate of 41.11%. A statistical analysis of the obtained data was performed with GraphPad Prism v.9. The results indicate that 55.1% of the community pharmacists have a good general knowledge and awareness regarding the off-label practice, although the legal frame is unclear. The responses highlight a high frequency of prescribing and request of medication for respiratory conditions (45.3%) and antibiotics (23.5%), with a concerning gap regarding the adverse events related to the off-label treatments (56.7%). A very small percentage of pharmacists (7.1%) contact a fellow healthcare professional when encountering an off-label prescription. In conclusion, in addition to the pharmacist’s conduct towards the best interest of the patient, there is a clear need to improve the doctor–pharmacist collaboration in order to make an off-label treatment successful.

## 1. Introduction

Medical personnel have the duty to prescribe the most suitable treatment for each patient, but when it comes to pediatric patients, this is often difficult to achieve. Thus, in order to prescribe a correct prescription from the point of view of the substance, the dose, the pharmaceutical form, and the route of administration, a doctor’s experience and knowledge can be doubled by the insights in pharmacology related to the drug, provided by the pharmacist [1]. A good collaboration between the experts in the fields of medicine and pharmacy can guarantee the best standard of care for pediatric patients.

Many medical prescriptions are outside the approved indication, and this practice is called “off-label”. The negative side of this practice is that many of the drugs prescribed off-label have no data to support this type of use [2]. It must be mentioned and understood that by resorting to off-label prescriptions, the doctor allows some space to treat a patient with few therapeutic options. However, this practice can lead to an increase in treatment costs, and a very important aspect is that it decreases the motivation to carry out trials evaluating off-label indications and to invest in order to obtain approval for a new use, if the profit does not exceed the amounts of money and time spent studying for approval [3,4,5]. Off-label practice is not illegal, many countries do not have legislation to prevent the use of off-label drugs, and the driving force behind this practice is the lack of suitable drugs for the patient [6,7]. For example, many European countries such as Italy, Spain, Germany, the Netherlands, Sweden, Greece, Hungary, the United Kingdom, and Lithuania approach the off-label use either through specific laws or through creating a regulatory frame in order to have a better control of the process [8]. France has a special approach for off-label situations, namely, a Temporary Recommendation for Use is issued by their national medicines agency (for more details, see [8]). The lack of a drug on the market for a certain age group does not automatically mean that the drug cannot be used in that age group. This is because regulatory bodies have not approved use for that age group due to a lack of evidence [9]. Also, from an ethical, moral, and professional point of view, the doctor who resorts to prescribing an off-label drug must have previously studied the data on off-label use, because otherwise he could be penalized for the malpractice of irresponsible use of off-label drugs [5,10,11]. In other words, off-label medication must be administered without hidden motives, only in the patient’s best interest and without fraudulent influences [9]. This is difficult to do because of the divergences that arise from countries’ policies [12].

Theoretically, most doctors and pharmacists are familiar with the notion of off-label, but the same cannot be said about patients. For this reason, patients should be informed whenever an off-label drug is prescribed, and should be counseled about the risks and benefits of the treatment [8].

Thus, a very important and perhaps often overlooked role is played by the dispensing pharmacist, who has the role of analyzing a medical prescription in an objective manner, having in mind the good interest of the patient. A pharmacists can determine whether the prescribed medication is suitable from several points of view (safety, effectiveness, legal framework, ethical considerations). To achieve results that protect the patient, both doctors and pharmacists must work together harmoniously, both entities having responsibility for treatment, the doctor for prescribing, the pharmacist for dispensing, acting as the last filter between treatment and patient [3].

Many of the drugs used off-label in children do not present pharmacokinetic data and data referring to dosage or type of formulation. For this reason, many drugs end up being used off-label. This happens not only for drugs with systemic administration, but also for those with topical administration, or with a modified route of administration [13,14,15,16,17].

Little is known about the awareness, the perceptions, and the attitude of community pharmacists towards off-label prescriptions. Most of the studies in the literature focus on off-label prescribing and dispensing of pediatric medication in hospital settings [18,19,20] rather than community pharmacies, although this phenomenon is very often encountered. Contrary to the hospital setting where an off-label treatment is strictly supervised and the potential adverse events could be rapidly and successfully managed, problems can arise in the community pharmacy segment where the patient does not benefit from strict medical supervision during the treatment. The pharmacist is the closest healthcare professional to the patient, availability-wise, thus he can explain, emphasize important details regarding the off-label treatment, and respond to patient’s concerns. Stewart D. et al. also recognize the key role of the community pharmacists in the prescribing–dispensing chain [21], hence the importance of their good knowledge and attitude in the matter. For this reason, we wanted to evaluate the level of knowledge and attitude of pharmacists regarding off-label drugs in the pediatric population. The results of this study help us to fill in the missing data in the literature on this topic and to improve educational programs with reference to off-label use, as well as to improve the safety of children.

## 2. Materials and Methods

### 2.1. Study Design, Setting, and Participants

A cross-sectional study was conducted between April and October 2022, for assessing the awareness and views of licensed pharmacists (community pharmacists) from Mureș county, Romania, regarding off-label drugs in the pediatric population. The study included both genders, and the sample size was estimated using the Raosoft software on the developer’s website. The required sample size was estimated at a 95% confidence level with an estimated 50% response distribution and with a margin of error of ±5%. The sample size was estimated as 218, and the final sample size was 236.

### 2.2. Questionnaire

The questionnaire was developed on the basis of previous studies [18,19,20,21,22,23,24]. A 28-item questionnaire was designed to evaluate the knowledge, awareness, views, and experience of community pharmacists in regard to off-label prescriptions and to reveal whether these are legal from their point of view. The questionnaire comprised five sections. The first section focused on general knowledge about off-label drug use and experience (4 items). The second section focused on the frequency of off-label prescriptions and dispensing (6 items). The third section focused on dispensing over-the-counter off-label drugs (5 items). The fourth section focused on off-label drug use without prescriptions (7 items). The fifth section focused on the awareness and views of community pharmacists on off-label practice (6 items). The content validity of the questionnaire was evaluated by a group of experts from our faculty. A pilot study was carried in order to see the clarity of the questions and modifications were made according to the feedback from the pilot test. On the first page, the objectives of the study were written along with the consent statement. The questionnaire was distributed via a website link on Google Forms and also on paper, which was provided personally by the interviewer. All pharmacists participated anonymously and voluntarily (Supplementary Material).

### 2.3. Statistical Analysis

Data were downloaded and manually introduced into Microsoft Excel software and analyzed in GraphPad Prism Software 16.89.1 (San Diego, CA, USA, v.9). Questionnaires with missing data or contradictory answers were excluded. Simple descriptive statistics (frequency and percentages) were used for various variables. Chi-square tests or Fisher’s exact tests were used where suitable. A *p* < 0.05 was considered statistically significant.

## 3. Results

The response rate for our questionnaire was 41.11%. No questionnaire was excluded, all of them meeting the inclusion criteria: community pharmacists. Exclusion criteria were missing data or contradictory answers. Only the most relevant questions for the present paper were included. We included a separate table for each specific section of the survey.

### 3.1. Experience and Knowledge about Off-Label Practice

Nearly half of the responses (46.2%) came from pharmacists with 1–5 years of experience (Table 1).

The majority of respondents stated that they have good knowledge about off-label practice (55.1%). Also, more than half responded that they became familiar with off-label practice in the university, which is expected (53.8%). However, a very small percentage is represented by professional training courses (7.6%), showing their necessity. Regarding the regulatory status, the majority of respondents said that off-label practice is legal, but not recognized (43.2%), when in fact, it is legal and recognized. Only 16.9% (40 participants) have knowledge of the regulatory status, 17 of them having 1–5 years of experience, 10 of them having 5–10 years of experience, 8 of them having 10–15 years of experience and 5 of them having more than 15 years of experience. This is expected, because in the last few years this subject was discussed more and more in the academic environment.

### 3.2. Frequency and Dispensing of Off-Label Prescriptions

When asked about the category of medication prescribed as off-label, the majority of respondents dispensed medication for acute respiratory conditions (45.3%), while dispensing based on indication and age accounts for two-thirds of all prescriptions. The most common reason for the prescriptions issued was the indication (32.4%), closely followed by age (31.8%). Almost all the pharmacists informed the parent or legal tutor about the off-label prescription or required the prescribers to note the reasons for off-label use, before dispensing the off-label medicines. This suggests that the pharmacists acknowledge an off-label prescription and are aware of the importance of it, of the risks, and also of the legal actions. Also, among those who contacted the prescribing doctor (73), 23 have experience between 1 and 5 years, 18 have experience between 5 and 10 years, 16 have experience between 10 and 15 years, and 16 have experience greater than 15 years. This indicates a tendency for doctor–pharmacist collaborations regarding prescription drugs (Table 2).

With respect to the question related to the adverse events that could appear, 8.4% stated that they know cases of adverse events consequent to an off-label treatment, while 56.7% declare that they do not have any knowledge regarding this matter. Despite the fact that a concern for safety and risks was observed, less than half (43.04%) of the respondents contacted the prescribing physician.

### 3.3. Dispensing Over-the-Counter Off-Label Drugs

In the third section, we noticed that the trend for medication for acute respiratory condition is maintained, this time 64.2% of the requests accounted for these kinds of medicines. The same trend followed for indication and age, as previously discussed. What is interesting to note is the attitude towards dispensing over-the-counter drugs meeting the off-label criteria. These attitudes show the awareness of the community pharmacists regarding the off-label medication, risks (adverse reactions, ineffectiveness), and also the legal actions (Table 3).

When questioned about over-the-counter medicines, the most common reason for requests was based on the indication (45.57%).

Among those who dispensed based on experience (30), 14 of them have experience between 1 and 5 years, 8 have experience between 5 and 10 years, 7 have experience between 10 and 15 years, and 1 has experience greater than 15 years. The percentage of those who consulted a doctor or a clinical pharmacist is small compared to the number of respondents equivalent to the same answer from Q10, showing the need to improve the doctor–pharmacist relationship. This must be done not only for prescription drugs but also for over-the-counter ones. Among those who resorted to collaboration with the doctor or clinical pharmacist (14), 4 have experience between 1 and 5 years, 5 have experience between 5 and 10 years, 4 have experience between 10 and 15 years, and 1 has experience greater than 15 years.

As in the previous section, regardless of whether the concern for risks and patient safety comes from experience or not, only 9.62% of pharmacists consulted a pediatrician/clinical pharmacist.

### 3.4. Dispensing Rx Off-Label Drug Use without Prescription

With regards to the fourth section, the requests for antibiotics represent more than one third from the total requests, as expected. Meanwhile, approximately 50% of the total requests fall into the off-label indication category, twice more than requests based on the age category. Refusal of dispensing the medicine in this case was almost three times higher than in the previous section (20.9% vs. 7.3%), showing a concern for patient safety and a ”better do no harm“ attitude. The fact that the doctor was not contacted was somehow justified, perhaps the best attitude in this case would have been to direct the patients towards the doctor (Table 4).

In case of antibiotic requests, most of the pharmacists searched for on-label alternatives (30%), and at a very close percentage are those who refused dispensing the medicine (28.75%). A total of 8.75% of the pharmacists dispensed the antibiotic based on their experience and 5% sought the advice of a pediatric specialist or a clinician pharmacist.

### 3.5. Views on Off-Label Practice

This section has a particularly important role because it shows the manner in which the pharmacists perceive off-label use, and also offers opportunities to improve the off-label practice. Regarding the situation in which the doctor must resort to such a practice, the answers are evenly distributed, each representing approximately one third. Regarding the benefits, the majority consider that it represents a way to respond to therapeutic needs. Regarding the best actions in the interest of patients, a positive attitude of pharmacists is observed, because they consider the existence of a database with information about off-label drugs, the continuous study, and the development of drugs to avoid off-label practice useful. Last but not least, for therapeutic success, the majority of respondents believe that there must be good communication between the doctor and the pharmacist, also emphasized in other answers (Table 5).

### 3.6. Comparison of Off-Label Familiarity between Pharmacists with Different Experience and Knowledge

In this section, we wanted compare the level of familiarization according to experience, in order to find a possible correlation of the experience versus knowledge regarding the legal frame for off-label use. The number of those who know the legal framework of off-label practice in Romania (40) is lower than the number of those who chose the other answers (196). It can also be observed that the number of those with knowledge and experience between 1 and 5 years is higher than the number of those with knowledge in the other categories of experience, possibly due to the fact that, in recent years, in the academic environment, off-label drugs have been discussed more (*p* = 0.0021). At the same time, the majority of respondents, regardless of age category, use the summary as sources of information, or in the case of those with 1–5 years of experience, specialized websites. It is observed that, of those who know the legal context of off-label practice (40), the majority consider that they have a good grasp of off-label notions. The same thing is observed in the case of people who do not know the legal context, the majority consider that they have a good grasp of the notions of off-label use (Table 6).

## 4. Discussion

In our study, the majority of the respondents are young pharmacists, with a professional experience from 1 to 5 years. These are expected numbers, due to the increased number of pharmacists in Romania, from 2018 to 2022. Also, this is due to the fact that the city is known for the powerful pharmaceutical market. Due to the fact that pharmaceutical education was not promoted in their time, the number of pharmacists from the other groups is smaller. The second reason, with a greater impact, is the reprofiling/change of job, due to low salaries from community pharmacies.

As expected, most respondents have heard about the concept of off-label use, but the degree of appreciation of the knowledge of this concept differs. Most have heard of this concept in an academic environment, especially those with less experience, between 1 and 5 years. It is also observed that many of the respondents do not know the legal framework of off-label practice, which is understandable because the legal framework is not clear. This can be improved by creating regulations and guidelines.

An interesting observation in this study is the prevalence of prescribing/dispensing of drugs for respiratory conditions. Both off-label medical prescriptions and OTC drugs for acute respiratory conditions represent a majority percentage (45.3% and 64.2%). These prescriptions include drugs that act at the bronchial level (mostly β_2_-stimulators and inhaled glucocorticoids). This is consistent with literature findings, where off-label prescribing of steroids and β_2_ agonists, dosage-wise, were identified in 90% of the responses [21] and off-label prescribing of antibiotics were also identified in almost all of the off-label categories [25].

Regarding the dispensing antibiotics without prescription, this is a global problem because it raises the issue of antibiotic resistance [26]. Contrary to the fact that this issue has been discussed for a long time, only a third of requests for antibiotics without a prescription are refused. The on-label search for an alternative could possibly indicate the fact that the pharmacist evaluated the patient condition as not requiring an antibiotic (e.g., a virosis) that could be managed with symptomatic treatment. Furthermore, a very small percentage of pharmacists liaise with a doctor or clinical pharmacist regarding antibiotic dispensing, following the same trend as in other studies, signaling a communication problem [21,27].

Although often overlooked, another very common problem that community pharmacists encounter on a daily basis consists in the request for off-label over-the-counter drugs. There are plenty OTC for pediatric use on the market [21] with an associated marketing advertising, and the parents resort to these without considering all of the aspects involved such as age, dosage, route of administration and so on. In our study, the most common off-label requests accounted for indication and age, indicating a strong need for input from the pharmacist’s side. As such, this section highlights the efforts of finding the best solution for the patient, whether it is finding an on-label alternative or dispensing the drug upon a detailed anamnesis combined with professional experience, with a very small percentage of pharmacists refusing the dispensing of the drug with no further action. Recently, the role of the pharmacist has undergone a transition, from the dispensing pharmacist, to the pharmacist who increasingly assumes responsibility for the medical act. This is difficult to notice or realize, because nowadays, in the society, this new role of the pharmacist is minimized, with them most of the time being considered a seller. From another perspective though, it can also be seen that very few pharmacists reach out to a fellow healthcare professional.

Altogether, Section 3 and Section 4 indicate a strong interest of the pharmacist to act towards a patient’s safety and their best interest, but also a strong need to improve the doctor–clinical pharmacist–community pharmacist relationship. In prescribing or dispensing off-label drugs, a patient’s safety can be enhanced through a concept that is as simple to understand as it is difficult to apply, namely doctor–pharmacist collaboration. The inclusion of the pharmacist in the medical record is accompanied by good results from the patients, which can be seen by a reduction in the number of hospitalizations [28,29]. These community pharmacist interventions can be based on medication management, patient counseling, and healthcare education [30]. In order to establish a strong partnership, pharmacists must demonstrate and apply their drug knowledge by providing useful information that results in improved patient health. However, such a collaboration where each member contributes through their knowledge is difficult to establish given the existing stereotypes in the medical field.

A 2013 Canadian study still applicable today shows that doctors want improved collaboration with pharmacists in order to enhance patient adherence and counseling. Instead, pharmacists want collaboration in medical decisions involving treatment choices such as drug interaction management. In the same study, two very important aspects are highlighted regarding the barriers that stand in the way of such collaborations. One is related to time, the second is related to the issue of risk management and the degree of responsibility assumed [31]. To these two aspects can be added the lack of communication or ineffective communication [32]. Also, the lack of effective collaborations is due to the lack of trust in specific skills or the diminishing of the importance of one of the participants [33]. As mentioned in another study, also seen in the present study, pharmacists rarely contact the doctor, thereby maintaining this lack of communication and collaboration [34]. In the present study, the desire to improve communication between doctors and pharmacists is shown, and also the desire for the creation of a database/guidelines containing information related to off-label medication.

An important finding is related to the occurrence of adverse events. A total of 53.2% of the respondents consider that the appearance of a potential acute adverse event is a risk associated with the off-label practice, yet only <10% declare that they have knowledge of this kind of events occurring due to an off-label treatment dispensed by them, while a concerning number of about 50% declared they do not have knowledge of it. This could be either because the patients never returned to the pharmacy with a feed-back, or because the pharmacist did not really make a close follow-up of the case. In reality, the literature reports a higher frequency of adverse reactions for off-label medication than for the on-label ones [35,36]. These studies are performed in hospital settings, where the treatments are closely monitored. In community pharmacy settings, the only possible monitoring would be through spontaneous reporting, which could be biased and subjective. However, it is a known fact that the adverse reactions related to off-label or unlicensed medicines are underreported [36]. A report from the European Medicines Agency indicated that the high prevalence of adverse reactions in pediatrics is linked to the off-label administration of antiasthmatics and antibiotics [36]. This is expected, as these drugs are used to treat the most common diseases in children. Considering the results in our study which also emphasize antiasthma and antibiotic medication as being the most prescribed/requested, we could extrapolate the same frequency of adverse events related to off-label treatment in non-hospitalized children, which is a serious concern. Nonetheless, as discussed in the previous paragraphs, it is of vital importance that the community pharmacist should closely monitor any off-label treatment, regardless of whether it is prescribed or requested by the patient themselves.

Community pharmacists share a common view on off-label treatment, most of them considering it a practice that fulfills unmet therapeutic needs and also that it should be practiced in the safest possible manner and throughout a good collaboration with the prescribing doctor. An aspect worth being discussed is related to the informed consent of the parents, given the fact that it is a very important component of the off-label practice. Although only a third of the pharmacists considered that the parental informed consent is to be obtained, previous sections indicated that when faced with an off-label prescription, a high percentage of the pharmacists informed the parents about the situation.

Despite the small number of correct responses regarding the legal status of off-label medication in Romania, the sixth and final section of the study reveals a strong correlation between the familiarity of the youngest practitioners, the knowledge of the legal framework, and the academic environment, where they learned about the off-label concept. This is a positive finding, meaning that the younger generations are trained for these kinds of prescriptions and dispensations, and that the managing of the off-label concept will be performed in a more consistent manner. As an improvement opportunity, there is a strong need for clarification around the legal status of off-label use, in order to ensure a safer practice. Regulatory bodies do not forbid this practice, nor do they encourage it. The Food and Drug Administration (FDA) supports the off-label use, in the sense that if a drug is administered other than specified in the Summary of Product Characteristics, the treatment and the outcome fall into the prescriber’s responsibility. In Europe, the European Commission makes it a purpose to ensure that medication is administered for its intended use, for the right population and age category. However, it allows the physician to prescribe an off-label treatment under their own responsibility, leveraging the access to treatment rather than restricting the medication where there are no other therapeutic options [8]. Even though the ideal concept would be to design medicines for every specific age and need, this goal is very hard to achieve, which is why the off-label concept remains the one practice that fulfills unmet therapeutical needs, and why healthcare professionals should be informed in order to make this practice as safe as possible.

The study has some limitations, due to the nature of the questionnaire study it is difficult to accurately determine whether the answers really represent the attitude of the respondents. However, due to the fact that this is a study that relied on anonymous responses, honesty was encouraged.

## 5. Conclusions

Off-label practice is an important component of the medical and pharmaceutical fields, with both positive and negative aspects. The key findings of our study consist in a good general awareness of the community pharmacists regarding the off-label practice, especially in the younger generation, and a positive attitude towards the attempt to make this practice as safe as possible, but also the need to clarify its legal status. We also identified some gaps throughout the study: one related to the adverse effects as consequences of off-label treatment, which for the time being are practically impossible to detect in any other manner than a close monitoring of the treatment by the community pharmacist. The other one is related to the lack of regulation of this practice which can impede a good doctor–pharmacist collaboration. An important idea emphasized throughout the study is the need to enhance the collaboration between these health professionals, in order to make off-label practice as safe as possible. Also, community pharmacists would benefit from trainings on the subject, especially those who did not have access to this information during their university courses. Until these aspects are improved, community pharmacists must use their expertise and make informed decisions towards the patient’s best interest.

## Figures and Tables

**Table 1:** Survey responses for the first section.

Question	Answer	Percentage (%)
Q1	1–5 years	46.2
	5–10 years	22.5
	10–15 years	16.5
	>15 years	14.8
Q2	Good	55.1
	A little bit	25.4
	Very well	18.2
	Not at all	1.3
Q3	Academic environment	53.8
	Experience	28.4
	Through professional colleagues	10.2
	Vocational training courses	7.6
Q4	Legal but unrecognized	43.2
	Illegal, but generally accepted	23.3
	Legal and recognized	16.9
	Illegal and unrecognized	16.5

**Table 2:** Survey responses for the second section.

Question	Answer	Percentage (%)
Q7	Medicines used in acute respiratory diseases	45.3
	Antibiotics	23.5
	Medicines used in the psychiatric field	21.8
	Others	9.4
Q9	Indication	32.4
	Age	31.8
	Dose	14.1
	Route of administration	12.4
	Pharmaceutical form	9.4
Q10	You have informed the parent/legal tutor	44.4
	You have contacted the prescribing doctor to request more information about the therapeutic choice made	43.2
	You dispensed the prescription without informing the prescriber	7.1
	You refused to dispense the prescription	3.6
	You dispensed the prescription without informing the parent/legal tutor	1.8
Q21	Yes No Don’t know	8.4 29.6 56.7

**Table 3:** Survey responses for the third section.

Question	Answer	Percentage (%)
Q12	Medicines used in acute respiratory diseases	64.2
	Antihistamines/other OTC substances with sedative properties, used to calm the child	30.5
	Others	5.3
Q14	Indication	45.7
	Age	35.1
	Dose	9.3
	Pharmaceutical form	5.3
	Route of administration	4.6
Q15	You searched for OTC, on-label therapeutic alternatives	37.1
	You have informed the parent/legal tutor that the request is for an off-label treatment	26.5
	You dispensed the medication based on your professional experience, following a careful anamnesis	19.9
	You have consulted a pediatrician or a clinical pharmacist in order to make an informed decision	9.3
	You have refused the dispensing of that medicine	7.3
Q22	Yes No Don’t know	9.3 25 51.7

**Table 4:** Survey responses for the fourth section.

Question	Answer	Percentage (%)
Q17	Antibiotics	35.1
	Medicines used in acute respiratory diseases	11.4
	Topical corticosteroids	7.6
	Medicines used in gastrointestinal disorders	7.2
	Antihistamines/other OTC substances with sedative properties, used to calm the child	5.5
	Others	2.1
Q19	Indication	49.4
	Age	24.1
	Dose	13.6
	Pharmaceutical form	8.0
	Route of administration	4.9
Q20	You have informed the parent/legal tutor that the request is for an off-label treatment	36.8
	You dispensed the medication based on your professional experience, following a careful anamnesis	23.3
	You searched for OTC, on-label therapeutic alternatives	12.9
	You have refused the dispensing of that medicine	20.9
	You have consulted a pediatrician or a clinical pharmacist in order to make an informed decision	6.1

**Table 5:** Survey responses for the fifth section.

Question	Answer	Percentage (%)
Q23	If his experience with that treatment is extensive and has been used successfully in several patients	36.6
	When the respective treatment is well documented in the literature or in specialist guidelines	32.3
	Only when other on-label therapeutic options have been ineffective	31.1
Q24	It meets the therapeutic needs in the pediatric sphere, as there are not enough medicinal substances/pharmaceutical forms for pediatric use	41.9
	It supports research in the field, as successful off-label treatments can be documented and serve as the scientific basis for approval of the indication for pediatric use	32.5
	It allows the doctor to act knowingly, in full professional capacity and experience, for the good of the patient	25.6
Q25	Occurrence of acute, potentially serious adverse effects, difficult to manage therapeutically	53.2
	The occurrence of unwanted long-term effects that could interfere with the normal development of the child	35.3
	Implications for the license to practice, as off-label can be associated with malpractice	7.2
	Violation of the patient’s right to benefit from a treatment that can be settled by the National Health Insurance House	4.3
Q26	Summary of product characteristics	43.8
	Specialty sites, such as DrugBank, Medscape, Drug List, etc.	30.6
	The drug leaflet	25.5
Q27	Existence of a database or guidelines containing information on off-label drugs	30.1
	An increased level of knowledge on the status of the drugs used in pediatrics by the pharmacist, information, continuous documentation through access to on-label therapeutic specialty courses for the total avoidance of off-label practice	25.0
	Development and approval of medicines for pediatric use, for the total avoidance of off-label practice	23.3
	Off-label practice cannot be totally eliminated	11.9
	Refusal to dispense the drug by the pharmacist and encourage the doctor to find alternatives	9.7
Q28	A good doctor–pharmacist collaboration, in order to optimize the treatment	38.1
	Informing parents about the respective treatment and obtaining their informed consent	33.1
	Treatment monitoring, documentation, and reporting of adverse reactions	26.3
	Informing the parents is not necessary, as it may cause them to become alarmed and refuse treatment, even though it may be the only therapeutic option for the child	2.5

**Table 6:** Comparison of off-label familiarity between pharmacists with different experience and knowledge.

	Familiar with the legal context of off-label practice in Romania				X^2^	*p*
	Yes		No			
1–5 years	17		92		0.80	0.847
5–10 years	10		43			
10–15 years	8		31			
>15 years	5		30			
	Circumstance of familiarity with off-label practice *					
	Academic environment	Professional experience	Vocational training courses	Through colleagues		
1–5 years	15	2	0	0	0.0021	
5–10 years	7	3	3	0		
10–15 years	3	5	1	1		
>15 years	1	4	0	2		
	Source of information					
	Summary	Leaflet		Sites		
1–5 years	54	21		34	6.723	0.347
5–10 years	19	13		10		
10–15 years	16	13		10		
>15 years	15	13		7		
	The degree of appreciation of knowing the notion of off-label in the legal context of the practice					
	Yes		No			
Very well	6		37		0.076	
Good	29		101			
A little bit	5		55			
Not at all	0		3			

* for those who know the legal context of off-label practice.

## Data Availability

The datasets that support the findings of this study are available from the first author upon reasonable request.

## Supplementary Materials

The following supporting information can be downloaded at: https://www.mdpi.com/article/10.3390/pharmacy12050149/s1, Questionnaires.
