# Supplementary material for: Off-Label Pediatric Medication Prescribing and Dispensing: Awareness and Attitudes among Community Pharmacists: A Questionnaire-Based Study
Source: Pharmacy (Basel). 2024 Sep 27;12(5):149. doi: 10.3390/pharmacy12050149 (PMC11511102; doi:10.3390/pharmacy12050149)
Supplement: Supplementary file 1 [file pharmacy-12-00149-s001.zip › pharmacy-3204753-supplementary.pdf]

Pediatric drugs and the "off-label" concept in pharmaceutical practice

1. Please specify your professional practice experience:
   a) 1-5 years
   b) 5-10 years
   c) 10-15 years
   d) >15 years
2. How well do you rate your knowledge of off-label medication?
   a) Good
   b) Very well
   c) A little bit
   d) Not at all
3. What was the circumstance in which you were introduced to this concept?
   a) Academic environment
   b) Experience
   c) Through professional colleagues
   d) Vocational training courses
4. To the best of your knowledge, the off-label practice in Romania is:
   a) Legal but unrecognized
   b) Legal and recognized
   c) Illegal, but generally accepted
   d) Illegal and unrecognized
5. In your daily practice, have you identified off-label pediatric drug prescriptions?
   a) Never
   b) Daily
   c) Weekly
   d) Monthly
6. If your answer to question 5 indicates that you encountered off-label prescriptions, what was their origin?
   a) Family physician
   b) The specialist doctor
7. If your answer to question 5 indicates that you encountered off-label prescriptions, what were the most commonly prescribed drug categories?

a) Medicines used in acute respiratory diseases
b) Antibiotics
c) Medicines used in the psychiatric field
d) Others

8. Could you provide specific examples of medicinal substances that reflect the answers to question 7?

9. If your answer to question 5 indicates that you have encountered off-label prescriptions, in which of the following off-label categories would you place those prescriptions, in terms of frequency?
   a) Indication
   b) Age
   c) Dose
   d) Route of administration
   e) Pharmaceutical form

10. If your answer to question 5 indicates that you encountered off-label prescriptions, what was your professional attitude?
    a) You have informed the parent/legal tutor
    b) You have contacted the prescribing doctor in order to request more information about the therapeutic choice
    c) You dispensed the prescription without informing the prescriber
    d) You refused to dispense the prescription
    e) You dispensed the prescription without informing the parent/legal tutor

11. 11. In your daily practice, have you identified situations where parents have requested OTC medications to be dispensed, that meet off-label criteria?
    a) Never
    b) Daily
    c) Weekly
    d) Monthly

12. If your answer to question 11 indicates that you encountered off-label requests, what were the most frequently requested drug categories?
    a) Medicines used in acute respiratory diseases
    b) Antihistamines/other OTC substances with sedative properties, used to calm the child
    c) Others

13. Could you provide concrete examples of drug substances that reflect the answers to question 12?

14. If your answer to question 11 indicates that you have encountered off-label requests, in which of the following off-label categories would you place those requests, in terms of frequency?
    a) Indication
    b) Age
    c) Dose
    d) Pharmaceutical form
    e) Route of administration
15. If your answer to question 11 indicates that you encountered off-label requests, what was your professional attitude?
    a) You searched for OTC, on-label therapeutic alternatives
    b) You have informed the parent/legal tutor that the request is for an off-label treatment
    c) You dispensed the medication based on your professional experience, following a careful anamnesis
    d) You have consulted a pediatrician or a clinical pharmacist in order to make an informed decision
    e) You have refused the dispensing of that medicine
16. In your daily practice, have you identified situations where parents have requested a prescription drug without having a prescription that meets off-label criteria?
    a) Never
    b) Daily
    c) Weekly
    d) Monthly
17. If your answer to question 16 indicates that you encountered off-label requests, what were the most frequently requested drug categories?
    a) Antibiotics
    b) Medicines used in acute respiratory diseases
    c) Topical corticosteroids
    d) Medicines used in gastrointestinal disorders
    e) Antihistamines/other OTC substances with sedative properties, used to calm the child
    f) Others
18. Could you provide concrete examples of medicinal substances that reflect the answers to question 17?

19. If your answer to question 16 indicates that you have encountered off-label requests, in which of the following off-label categories would you place those requests, in terms of frequency?
    a) Indication
    b) Age
    c) Dose
    d) Pharmaceutical form
    e) Route of administration
20. If your answer to question 16 indicates that you encountered off-label requests, what was your professional attitude?
    a) You have informed the parent/legal tutor that the request is for an off-label treatment
    b) You dispensed the medication based on your professional experience, following a careful anamnesis
    c) You searched for OTC, on-label therapeutic alternatives
    d) You have refused the dispensing of that medicine
    e) You have consulted a pediatrician or a clinical pharmacist in order to make an informed decision
21. Have you encountered situations in which, following the dispense based of an off-label prescription, the patient experienced adverse reactions?
    a) Yes
    b) No
    c) Don't know
22. Have you encountered situations in which, following the dispense of an OTC medication off-label, the patient experienced adverse reactions?
    a) Yes
    b) No
    c) Don't know
23. When do you think a doctor should resort to off-label prescribing?
    a) When his experience with that treatment is extensive and has been used successfully in several patients
    b) When the respective treatment is well documented in the literature or treatment guidelines
    c) Only when other on-label therapeutic options have been ineffective

24. What do you consider to be the benefits associated with off-label practice in pediatrics?
    a) It meets the therapeutic needs in the pediatric sphere, as there are not enough medicinal substances/pharmaceutical forms for pediatric use
    b) It supports research in the field, as successful off-label treatments can be documented and serve as the scientific basis for approval of the indication for pediatric use
    c) It allows the doctor to act knowingly, in full professional capacity and experience, for the good of the patient
25. What do you think are the risks associated with off-label practice in pediatrics?
    a) Occurrence of acute, potentially serious adverse effects, difficult to manage therapeutically
    b) The occurrence of unwanted long-term effects that could interfere with the normal development of the child
    c) Implications for the license to practice, as off-label can be associated with malpractice
    d) Violation of the patient's right to benefit from a treatment that can be settled by the National Health Insurance House
26. What are your sources of information when it comes to pediatric drugs?
    a) Summary of product characteristics
    b) The drug leaflet
    c) Specialty sites, such as DrugBank, Medscape, Drug List, etc.
    d) Other sources; please specify _______
27. Considering that off-label practice is a large-scale phenomenon in the pediatric sphere, what do you think would be the most appropriate actions in the patient's interest?
    a) Existence of a database or guidelines containing information on off-label drugs
    b) An increased level of knowledge on the status of the drugs used in pediatrics by the pharmacist, continuous information and documentation through access to specialty courses
    c) Development and approval of medicines for pediatric use, for the total avoidance of off-label practice
    d) Off-label practice cannot be totally avoided

e) Refusal to dispense the drug by the pharmacist and encourage the doctor to find alternatives

28. Considering that off-label practice is a large-scale phenomenon in the pediatric sphere, which cannot be completely eliminated due to unmet therapeutic needs, in your opinion, what would be the most appropriate actions to achieve therapeutic success?
    a) A good doctor-pharmacist collaboration, in order to optimize the treatment
    b) Informing parents about the respective treatment and obtaining their informed consent
    c) Treatment monitoring, documentation and reporting of adverse reactions
    d) Informing the parents is not necessary, as it may cause them to become alarmed and refuse treatment, even though it may be the only therapeutic option for the child
